# Physiological Barriers to Nucleic Acid Therapeutics and Engineering Strategies for Lipid Nanoparticle Design, Optimization, and Clinical Translation

**DOI:** 10.3390/pharmaceutics17101309

**Published:** 2025-10-08

**Authors:** Yerim Kim, Jisu Park, Jaewon Choi, Minse Kim, Gyeongsu Seo, Jeongeun Kim, Jeong-Ann Park, Kwang Suk Lim, Suk-Jin Ha, Hyun-Ouk Kim

**Affiliations:** 1 Division of Chemical Engineering and Bioengineering, College of Art, Culture and Engineering, Kangwon National University, Chuncheon-si 24341, Gangwon-do, Republic of Korea; 03erinkim@kangwon.ac.kr (Y.K.); qkrwltn0905@kangwon.ac.kr (J.P.); choijw8839@kangwon.ac.kr (J.C.); minse0415@kangwon.ac.kr (M.K.); x6199x@kangwon.ac.kr (G.S.); 202112527@kangwon.ac.kr (J.K.); kslim@kangwon.ac.kr (K.S.L.); sjha@kangwon.ac.kr (S.-J.H.); 2 Institute of Fermentation of Brewing, Kangwon National University, Chuncheon-si 24341, Gangwon-do, Republic of Korea; 3 Department of Smart Health Science and Technology, Kangwon National University, Chuncheon-si 24341, Gangwon-do, Republic of Korea; 4 Department of Environmental Engineering, College of ACE, Kangwon National University, Chuncheon-si 24341, Gangwon-do, Republic of Korea; pjaan@kangwon.ac.kr

**Keywords:** lipid nanoparticles, nucleic acid therapeutics, physiological barriers, endosomal escape, targeted delivery

## Abstract

Lipid nanoparticles are a clinically validated platform for delivering nucleic acids, but performance is constrained by multiscale physiological barriers spanning circulation, vascular interfaces, extracellular matrices, cellular uptake, and intracellular trafficking. This review links composition–structure–function relationships for ionizable lipids, helper phospholipids, cholesterol, and PEG-lipids to systemic fate, endothelial access, endosomal escape, cytoplasmic stability, and nuclear transport. We outline strategies for tissue and cell targeting, including hepatocyte ligands, immune and tumor selectivity, and selective organ targeting through compositional tuning, together with approaches that modulate escape using pH-responsive chemistries or fusion-active peptides and polymers. We further examine immunomodulatory co-formulation, route and schedule effects on biodistribution and immune programming, and manufacturing and stability levers from microfluidic mixing to lyophilization. Across these themes, we weigh trade-offs between stealth and engagement, potency and tolerability, and potency and manufacturability, noting that only a small fraction of endosomes supports productive release and that protein corona variability and repeat dosing can reshape tropism and clearance. Convergence of standardized assays for true cytosolic delivery, biomarker-guided patient selection, and robust process controls will be required to extend LNP therapeutics beyond the liver while sustaining safety, access, and scale.

## 1. Introduction

Lipid-based nanoparticles have emerged as a clinically validated platform for delivering diverse nucleic acids, including siRNA, mRNA, and plasmid DNA [1,2]. Their modular composition—ionizable lipids, helper phospholipids, cholesterol, and PEG-lipids—enables scalable manufacturing and tunable interactions with biological systems [2,3]. Despite these strengths, translational performance is constrained by multiscale barriers spanning circulation and endothelial interfaces to extracellular matrices, cellular uptake, endosomal escape, cytoplasmic stability, and, for DNA payloads, nuclear access [4]. Practical design must balance stealth with target engagement and reconcile potency with tolerability and manufacturability while accounting for protein corona dynamics and the reality that only a small subset of endosomes supports productive cytosolic release [5,6].

A physiological viewpoint helps organize these constraints. In the bloodstream, hemodynamic shear, dilution, and opsonization reshape nanoparticle identity and clearance by the mononuclear phagocyte system [5]. Tissue entry depends on organ-specific vascular phenotypes, interstitial pressure, and matrix architecture that jointly regulate extravasation and penetration [4]. At the cellular scale, uptake routes are heterogeneous and often deliver cargo to degradative compartments, which underscores the narrow operational window for pH-responsive ionizable lipids, helper lipid–cholesterol tuning, and PEG deshielding to enable endosomal escape [7]. Route and schedule of administration further modulate biodistribution and immune programming, and manufacturing levers such as microfluidic mixing and lyophilization determine reproducibility and long-term stability across use cases [4].

In this review paper, we synthesize composition–structure–function relationships that govern systemic fate and intracellular trafficking and map them to engineering strategies that improve the delivery of nucleic acids with an emphasis on translational practicality. We first outline the roles and trade-offs of ionizable lipids, helper phospholipids, cholesterol, and PEG-lipids and the physicochemical properties that influence morphology, size, and surface potential [1,2]. We then examine organ and cell targeting, including ligand-guided approaches and selective organ targeting via compositional tuning, alongside mechanisms and enhancers of endosomal escape and considerations for cytoplasmic and nuclear delivery [4,6]. Finally, we discuss immunomodulatory co-formulation, route and dosing strategies, and manufacturing and stability controls, highlighting the need for standardized assays that quantify true cytosolic delivery and for process rigor that links formulation to clinical performance [4,5]. In doing so, this review moves beyond a descriptive summary by introducing a barrier-by-barrier engineering framework, providing a critical perspective on why certain strategies succeed or fail, and outlining a structured roadmap for rational LNP design across different biological obstacles. To illustrate this framework in a concise manner, the major barriers, corresponding challenges, and representative strategies are systematically summarized in Table 1.

## 2. Composition, Physicochemical Properties, and Encapsulation of Lipid-Based Nanoparticles

### 2.1. Core Components and Functional Roles of Lipid-Based Nanoparticles

Lipid-based nanoparticles for nucleic acid delivery comprise an ionizable lipid, a helper phospholipid, cholesterol, and a PEG-lipid, and their proportions and chemistries govern stability, pharmacokinetics, uptake, and endosomal release [8]. Ionizable lipids are neutral in blood and protonate in endosomes; head-group pKa, tail architecture, and linkers tune phase behavior and membrane disruption for cytosolic entry, but overly active designs can increase hemolysis or inflammation [9]. Helper phospholipids maintain bilayer integrity; highly ordered species impede fusion, whereas unsaturated variants aid fusion at the cost of physical stability [10]. Cholesterol adjusts fluidity and packing, with moderate levels favoring nonlamellar transitions and excessive amounts stiffening membranes and shifting tropism [11]. PEG-lipids provide steric shielding and extend circulation, but high density or slow shedding suppress uptake and escape, so chain length, fraction, and deshielding kinetics require tuning to route and tissue [12]. Optimal composition is context-specific and must balance efficacy, manufacturability, and safety. Table 2 summarizes roles and limitations.

#### 2.1.1. Ionizable Lipids

Ionizable lipids enable cytosolic delivery through pH-triggered charge switching, promoting endosomal membrane disruption while remaining neutral in blood to limit nonspecific interactions [13]. Previous studies report that head-group chemistry, apparent pKa, tail saturation and branching, and linker architecture govern packing, phase behavior, and formation of fusogenic or nonlamellar states [14]. Formulations with higher apparent pKa can increase serum binding and clearance, whereas low values reduce endosomal escape, so effective ranges depend on composition [13]. Overly disruptive motifs raise risks of hemolysis and inflammation, so designers must balance escape potency with safety [15]. Rational tuning should couple pKa with lipid ratios and PEG-lipid shedding kinetics for route [16].

#### 2.1.2. Helper Phospholipids

Helper phospholipids maintain lamellar structure, reduce leakage, and modulate fusion in lipid-based nanoparticles [11,17]. Previous studies report that high melting species such as DSPC increase rigidity, resist serum-induced destabilization, and improve stability, but can impede membrane fusion and slow endosomal release [11,18]. More unsaturated or shorter chain phospholipids promote fusogenic transitions and cargo release, although they compromise physical stability [17]. The molar ratio of ionizable lipids to cholesterol shapes phase behavior, affecting protein corona formation, biodistribution, and organ tropism [18]. Excess helper content can dilute the ionizable component and depress endosomal escape. Selection should couple stability targets with the intended route and storage constraints [11].

#### 2.1.3. Cholesterol

Cholesterol affects the packing, fluidity, and phase behavior of lipid-based nanoparticles, which in turn influences their stability in blood and the probability of endosomal escape [19]. Prior research demonstrates that intermediate fractions promote fusogenic nonlamellar states and reduce leakage, thus improving cytosolic delivery efficiency [20]. Elevated cholesterol levels enhance membrane rigidity, decrease fusion efficiency, and may modify organ tropism by affecting particle deformability and the composition of the protein corona [21]. The interactions of ionizable lipids with helper phospholipids create a balance between lamellar and hexagonal phases, while the chain length and saturation of neighboring lipids affect the organization of cholesterol within the bilayer. Oxidized derivatives and variability in batches pose risks to stability and safety. The formulation seeks to establish equilibrium between circulation durability and endosomal release [22].

#### 2.1.4. PEG-Lipids

**Figure 1:**
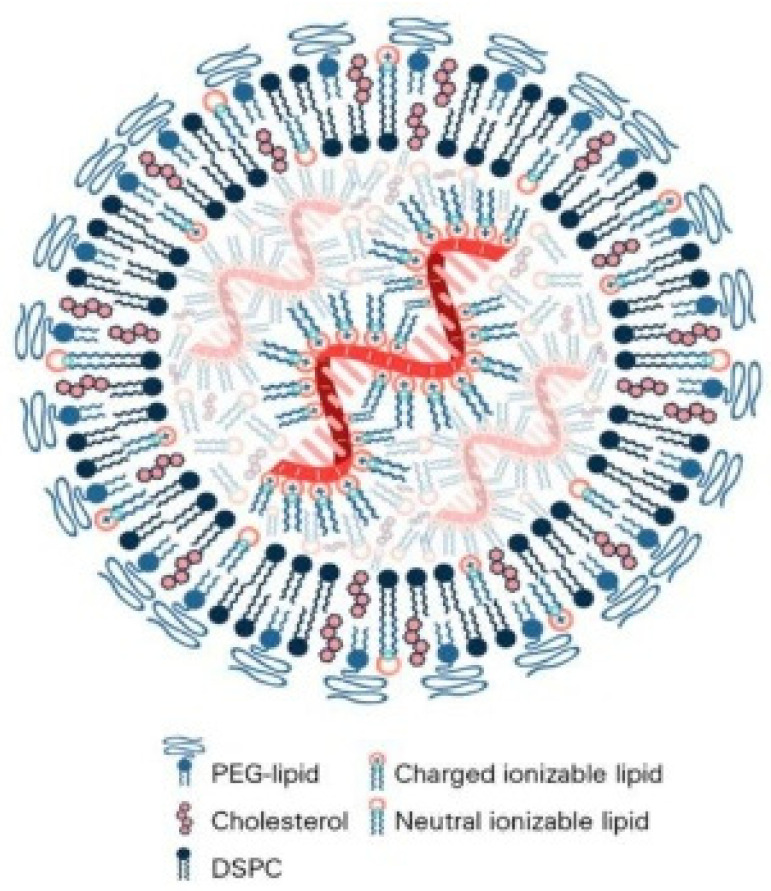
Schematic of a lipid-based nanoparticle encapsulating nucleic acids. The typical formulation contains an ionizable lipid, a PEG-lipid, a helper phospholipid such as DSPC, and cholesterol. Ionizable lipids switch charge in endosomes to aid escape, helper phospholipids and cholesterol organize the bilayer and can promote fusion when properly tuned, and PEG-lipids provide transient steric shielding during circulation.

PEG-lipids provide steric shielding that reduces opsonization and uptake by the mononuclear phagocyte system, extending circulation while potentially masking ligands and slowing endosomal release [18]. Previous studies report that chain length, surface density, and anchor hydrophobicity control deshielding kinetics and protein corona composition, which govern cellular uptake and escape [23]. High surface coverage or slowly shedding anchors depress delivery, whereas insufficient coverage promotes aggregation and rapid clearance [24]. Immune responses against PEG and accelerated clearance on repeat dosing remain concerns [25]. Effective designs pair modest PEG fractions with cleavable or short anchors coordinated with ionizable lipid content, route, and target tissue. As illustrated in Figure 1, PEG affords transient stealth during circulation while helper phospholipids and cholesterol organize the bilayer and ionizable lipids drive endosomal escape [18]. PEG-lipids are becoming more linked to anti-PEG immune responses, which may speed up blood clearance (ABC) and help keep blood in circulation for longer [26]. One major problem with translating research into practice, especially for long-term medications, is that repeated doses make them much less effective [26]. This is due to treatment-induced or pre-existing anti-PEG antibodies, according to clinical results [27]. PEGylation is a stabilizing method, but it also has a risk that could make it less effective in the long run [28].

### 2.2. Physicochemical Properties of Lipid-Based Nanoparticles

#### 2.2.1. Particle Size and In Vivo Fate

Particle size governs dispersion, clearance, and tissue access of lipid-based nanoparticles. Previous studies report that hydrodynamic diameters around 60–120 nm favor prolonged circulation and extravasation, whereas particles below 50 nm undergo rapid renal filtration and those above 200 nm are preferentially sequestered by the mononuclear phagocyte system [29]. Size also modulates lymphatic trafficking after subcutaneous dosing and influences uptake routes and endosomal escape. A single universal optimum is unreliable because endothelial phenotype and enhanced permeability and retention vary by tissue. Effective design controls mean size and polydispersity while anticipating postinjection changes from protein corona formation and aggregation [30]. Reported data should distinguish hydrodynamic and dry diameters and verify stability in serum and under shear so choices align with route and target organ [31].

#### 2.2.2. Morphological Architecture

Lipid-based nanoparticles are often labeled spherical, but composition, mixing kinetics, and payload generate diverse internal architectures including solid cores, inverted micelles, and multilamellar vesicles [20]. Previous studies report that morphology dictates nucleic acid distribution, water content, and phase behavior, which in turn modulate fusion propensity, endosomal escape, and release kinetics [20]. Non-spherical or multilamellar structures can improve stability in blood while slowing cytosolic delivery, whereas loosely packed cores accelerate leakage. Morphology is sensitive to ionizable lipid ratio, cholesterol fraction, buffer and pH during mixing, and can transition under endosomal conditions [32]. Assignments based only on negative stain imaging are unreliable, so characterization should use cryo electron microscopy, tomography, and complementary scattering with explicit reporting of buffers and shear. Design should target forms that balance shelf stability, on-target release, and manufacturability [33].

#### 2.2.3. Surface Charge

**Figure 2:**
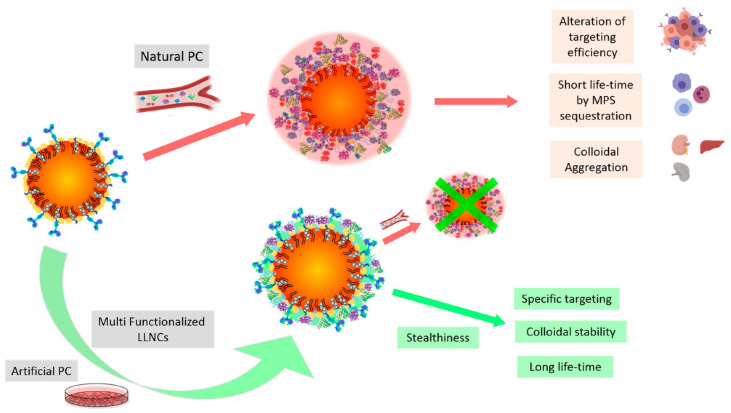
Surface potential and zeta potential of lipid nanoparticles. Schematic showing ion distribution, Stern layer, and slipping plane. The resulting zeta potential governs serum stability, protein corona formation, aggregation, and cellular interactions.

Surface potential governs serum stability, protein corona formation, aggregation, and cellular interactions of lipid-based nanoparticles, and its interpretation should account for morphology and assay conditions, as shown in Figure 2 [34]. Near-neutral or slightly negative zeta potential limits nonspecific binding and supports longer circulation, whereas strongly positive surfaces increase complement activation, hemolysis risk, and rapid clearance despite enhanced adhesion [35]. Apparent neutrality can suppress uptake and endosomal escape if charge switching is insufficient, and PEG-lipids may mask charge in blood and therefore should be shed at the target [35]. Reported values shift with buffer, ionic strength, pH, temperature, and adsorbed proteins and are sensitive to polydispersity [36]. Rigorous characterization includes electrophoretic mobility with explicit medium composition and verification under physiologic serum and shear [37]. Formulation should coordinate ionizable lipid content, PEG density and anchor length, and buffer selection to balance safe circulation with timely cellular entry.

### 2.3. Nucleic Acid Encapsulation Mechanisms

Efficient entrapment arises during rapid mixing of an acidic aqueous nucleic acid stream with an ethanolic lipid stream, which protonates ionizable lipids and drives electrostatic complexation that nucleates particle assembly [35]. Previous studies report that cargo partitions to the interior as ion pairs within water-rich or nonlamellar domains whose organization depends on composition and mixing history [36]. Key levers include pH relative to the apparent pKa, the N-to-P ratio, solvent fraction, ionic strength, temperature, and both flow rate and flow-rate ratio during mixing [17]. Increasing charge density raises encapsulation and can enlarge particles or promote aggregation, whereas insufficient charge lowers loading and diminishes endosomal escape [38]. PEG-lipid fraction and anchor length set deshielding kinetics and influenced cargo distribution by controlling kinetic trapping versus reorganization [37]. Scalable microfluidic mixing improves batch consistency, but buffer exchange and dilution sequences reshape morphology [38]. Formulation should balance loading, size control, and release while documenting conditions for reproducibility [38].

## 3. Systemic Fate and Biological Barriers of Lipid-Based Nanoparticles

### 3.1. Hemodynamic Shear and Dilution in the Bloodstream

Intravenous administration induces hemodynamic shear and convective dilution, which promptly decreases local nanoparticle concentration and reduces their residence time near the endothelium, as detailed in Table 3 [39]. High shear conditions can enhance PEG desorption, alter the protein corona, and facilitate aggregation [40]. These effects are exacerbated by increased hydrodynamic size or a strong positive surface potential, ultimately compromising stability and extravasation [39]. The initial mixing is influenced by the injection rate, catheter geometry, and infusion site, which can subsequently alter downstream morphology. Mitigation is most effective with hydrodynamic diameters ranging from 60 to 120 nm, exhibiting near-neutral surface potential, and utilizing moderate PEG with cleavable or short anchors to facilitate subsequent deshielding [38]. Adjusting viscosity or implementing slower infusion techniques decreases peak shear; however, this may constrain dosing logistics. Performance must be assessed under physiological serum conditions, ionic strength, and flow dynamics. Designs should achieve a balance between circulation durability and endothelial engagement to prevent excessive shielding.

### 3.2. Protein Corona and Opsonization

**Figure 3:**
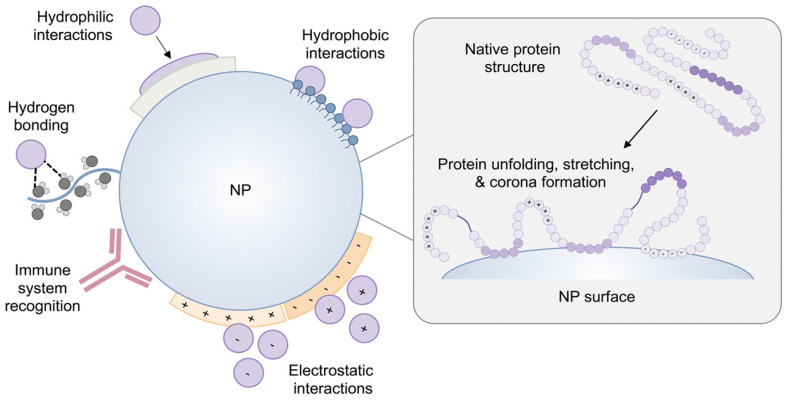
Schematic illustration of protein corona formation on nanoparticles. Upon exposure to biological fluids, nanoparticles rapidly acquire adsorbed protein layers consisting of a hard and soft corona, which redefine their biological identity and influence opsonization, immune clearance, circulation time, biodistribution, and delivery efficiency.

Upon contact with blood, lipid-based nanoparticles rapidly acquire adsorbed protein layers that reshape surface potential, hydrodynamics, and biological identity, thereby governing circulation, biodistribution, and cellular interactions (Figure 3) [27]. Opsonins such as immunoglobulins and complement promote recognition by liver and spleen macrophages, lowering on-target delivery and increasing the risk of immune activation [41]. Corona composition depends on size, surface potential, lipid composition, PEG-lipid density, and shear [42]. Antifoul coatings reduce nonspecific adsorption but do not eliminate it, and apolipoproteins can still associate and redirect trafficking [43]. Excessively inert surfaces may suppress uptake and endosomal escape, so design favors-controlled corona formation through PEG tuning, zwitterionic or biomimetic interfaces, or preconditioning strategies [6]. Experimental assessments should use matched serum and physiologic flow to capture the evolution of soft and hard layers [44].

#### Challenges and Opportunities of Protein Corona

One of the central paradoxes in lipid nanoparticle (LNP) delivery is that, even when particles are decorated with ligands for active targeting, the adsorbed protein corona can cover these ligands and ultimately determine biological fate [30,42,44]. This masking effect weakens receptor-mediated targeting and remains a major challenge for clinical translation [45]. Although strategies such as PEGylation, zwitterionic modification, or bioinspired coatings reduce nonspecific protein adsorption, they do not fully eliminate corona formation. In fact, excessive shielding can hinder cellular uptake and limit endosomal escape, creating a delicate balance between circulation stability and target engagement [42,45]. Interestingly, certain corona constituents, including apolipoprotein E, may promote liver-specific delivery, raising the possibility that corona modulation—rather than complete suppression—could be leveraged to redirect biodistribution [43]. To move the field forward, systematic studies are required to characterize corona “fingerprints,” identify the conditions under which ligand masking is most problematic, and test new approaches such as pre-coating or controlled corona engineering [46]. Given that corona composition is highly dynamic and varies between patients, resolving this paradox will be critical for achieving consistent and reproducible outcomes with LNP-based nucleic acid therapeutics [44,47].

### 3.3. Reticuloendothelial System (RES) Clearance

Following intravenous administration, lipid-based nanoparticles swiftly interact with macrophages in the reticuloendothelial system located in the liver, spleen, and lymph nodes, with Kupffer cells frequently seizing a substantial proportion within minutes, thereby restricting extrahepatic exposure [48]. Prior research indicates that clearance correlates with hydrodynamic size, surface potential, deformability, and protein corona composition, with particles exceeding 150 nm or possessing strongly cationic surfaces being preferentially internalized, while opsonins enhance recognition [49]. Repeated administration can elicit anti-PEG reactions that reduce circulation time and alter trafficking pathways [50]. Mitigation integrates sub-150 nm dimensions, near-neutral surfaces, calibrated PEG fractions with cleavable or short anchors, zwitterionic or biomimetic interfaces, and decoy or preconditioning strategies [4]. Excessive shielding may hinder ligand binding and endosomal release; therefore, formulations must equilibrate stealth with target engagement and validate efficacy in physiological serum, flow, and species-appropriate models [4].

#### PEG Stealth Effect and Emerging Immunological Concerns

**Figure 4:**
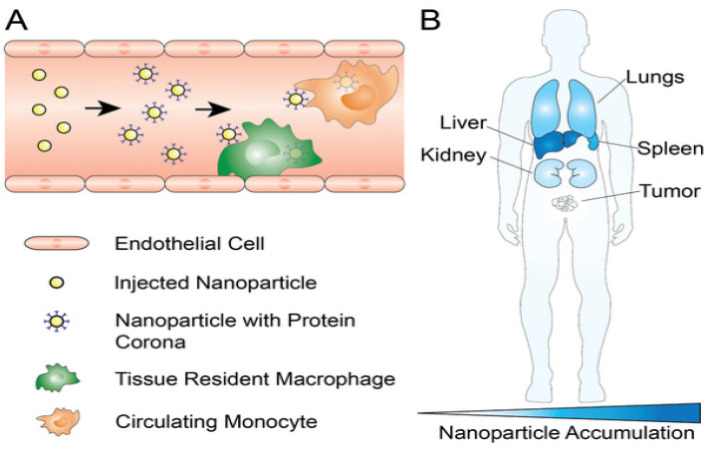
Schematic comparison of vascular endothelial structures that influence lipid nanoparticle delivery. (**A**) Intravascular events showing nanoparticles acquiring a protein corona and interacting with endothelium and tissue macrophages (**B**) organ-level tendencies for accumulation in liver, spleen, tumors, lungs, and kidney. Taken from [53] with permission.

PEGylation has long been employed to prolong the circulation of lipid nanoparticles, yet the emergence of anti-PEG antibodies and the accelerated blood clearance (ABC) phenomenon continue to represent major barriers to clinical translation. Upon repeated dosing, both IgM and IgG responses can be elicited against PEG, which promotes opsonization, rapid removal from the bloodstream, and a significant decline in therapeutic performance. These issues are especially problematic for chronic indications where multiple administrations are required over time [51]. To mitigate such challenges, attention has shifted toward alternative stealth materials including poly(2-oxazoline), poly(glycerol), polysarcosine, and zwitterionic polymers, each of which has shown the potential to maintain circulation benefits with lower immunogenicity [28,52]. It is also becoming increasingly clear that the “stealth effect” is not an absolute property but depends strongly on PEG density, chain length, and conformational arrangement. Recent analyses highlight that striking the right balance between extended circulation and efficient cellular uptake will be critical for achieving successful translation of LNP-based therapeutics [51]. These circulation and clearance dynamics, along with organ-level biodistribution patterns, are illustrated in Figure 4.

### 3.4. Tissue-Specific Vascular Endothelial Barriers

Transvascular transport is dictated by organ-specific endothelium, as illustrated in Figure 4 and summarized in Table 4. Liver sinusoids are fenestrated and lack a continuous basement membrane, with dynamic pores typically around 50–200 nm arranged in sieve plates, which permit passage of small and deformable particles while Kupffer cells rapidly curtail parenchymal access [54]. The spleen presents interendothelial slits that act as mechanical filters and favor retention of rigid or larger particles, so a size near 60–120 nm and adequate deformability are advantageous [55]. Most healthy tissues display continuous tight junctions and a thick glycocalyx that restrict passive flux, which shifts emphasis to receptor-mediated transcytosis through targets such as transferrin or scavenger receptors and to careful control of surface stealth to allow endothelial engagement. Tumors show disorganized and leaky vessels with pore cutoffs that vary widely across lesions and patients, supporting enhanced permeability and retention while high interstitial pressure, slow flow, and dense extracellular matrices limit deep penetration [56,57]. The kidney glomerulus is fenestrated but the basement membrane and slit diaphragm exclude nanoparticles, and the lung capillary bed combines thin endothelium with tight junctions and high shear that disfavor passive extravasation [58]. Effective design aligns size, deformability, and ligand choice with the local phenotype and dosing route and verifies performance under physiological shear and serum.

## 4. Microenvironmental and Cellular Barriers to Lipid Nanoparticle Delivery

### 4.1. Extracellular Matrix Constraints on Nanoparticle Penetration

**Figure 5:**
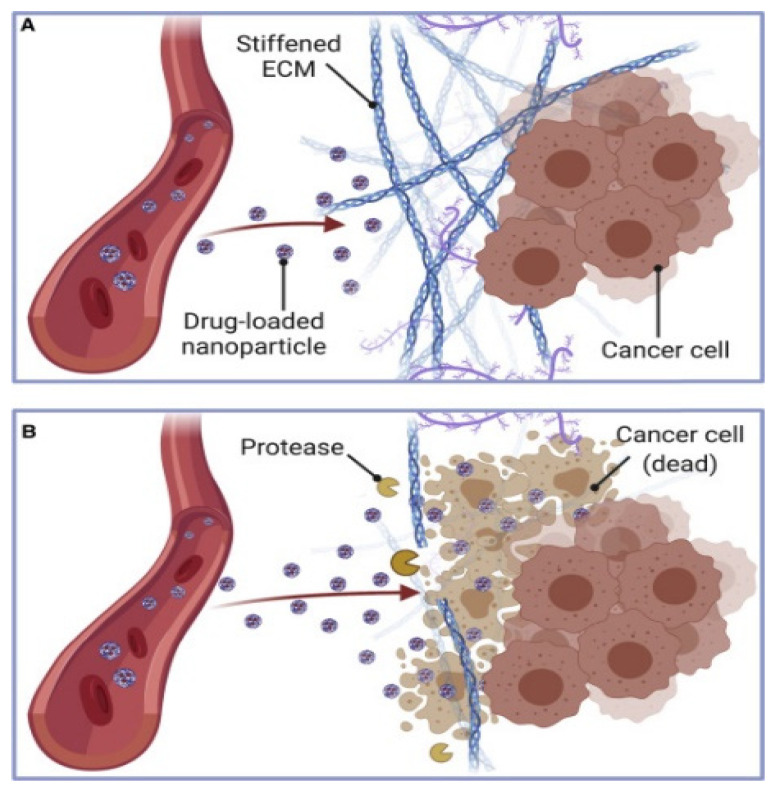
Tumor extracellular matrix as a barrier to lipid nanoparticle delivery of nucleic acids. (**A**) Dense and stiff matrix limits the penetration of drug-loaded nanoparticles. (**B**) Proteolytic remodeling by matrix metalloproteinases, hyaluronidases, or collagenases relaxes the matrix and improves delivery. Taken from [64] with permission.

The extracellular matrix in solid tumors forms a dense, cross-linked network of collagen, hyaluronic acid, and proteoglycans that narrows interstitial pores to about 20–60 nm and increases stiffness, creating tortuous paths that impede lipid-based nanoparticles carrying siRNA or mRNA when sizes exceed roughly 30–40 nm [59]. Negatively charged glycosaminoglycans can electrostatically trap cationic carriers, producing local accumulation and uneven distribution, while fibroblast activation and collagen cross-linking further compact the stroma and diminish convective transport [60]. Nanoparticles face increased challenges in navigating heterogeneous microenvironments [61]. This happens because the extracellular matrix is being remodeled in an unusual way, stromal cells are being activated, and fibroblasts are being linked to cancer [62]. Recent studies show that restrictions caused by the matrix and cells make it hard for the drug to spread evenly, which leads to different therapeutic results for each patient [63]. Approaches under evaluation include hydrophilic stealth shells such as PEG or zwitterionic coatings to limit matrix binding, adaptive designs that shrink or neutralize charge within the interstitium [55], coadministration of matrix-remodeling enzymes including collagenase, hyaluronidase, or MMP mimetics, and physical modalities such as high-intensity focused ultrasound or mild hyperthermia to transiently relax the matrix as depicted in Figure 5 [59]. Each option involves trade-offs because steric shielding can lower cellular uptake, enzymatic remodeling can harm healthy matrix and offer only a brief permeability window, and device-assisted methods can yield spatially uneven exposure within heterogeneous tumors [59]. Coordinated regimens that time LNP dosing with a short remodeling window and carriers engineered for reversible charge exposure after penetration provide a pragmatic path to deeper transport with preserved intracellular delivery efficiency [59].

#### ECM-Associated Stability and Storage

A recent study demonstrated that the biophysical density and heterogeneity of the extracellular matrix (ECM), in conjunction with the physicochemical stability of nanoparticle formulations, influence penetration and treatment effects [65]. A recent study of extracellular vehicles (EVs) has shown that storage conditions and their surroundings affect the recovery, integrity, and functional activity of these vesicles [18]. Lipid-based nanoparticles (LNPs) can become less stable, and their interactions can be deformed upon exposure to heterogeneous extracellular matrix (ECM) networks in vivo [18]. Under adverse conditions, protein adsorption, vesicle fusion, or aggregation can enhance complex transport pathways inside collagen- and glycosaminoglycan-rich matrices [30]. Therefore, the integration strategy should consider both ECM remodeling (e.g., enzyme or physical modulation) and formulation stabilization (e.g., optimized buffer system, cryoprotectant, or dynamic coating) to preserve functional delivery [64]. This dual perspective that connects extracellular transport barriers with physicochemical conservation gives a complete framework for making nanomedical devices that can be used in medicine [18].

### 4.2. Interstitial Fluid Pressure and Transport Barriers

Solid tumors are often described through the lens of the enhanced permeability and retention effect, but the expected convective entry of lipid nanoparticles carrying nucleic acids is curtailed by elevated interstitial fluid pressure that flattens transvascular gradients and drives outward fluid flow, leaving payloads stranded near vessels rather than within the core [66,67]. High pressure arises from a hyperdense extracellular matrix, leaky and compressed vessels, and poor lymphatic drainage, which together impede transport of liposomes and LNPs, skew intratumoral distribution, and support phenotypes linked to invasion and immune evasion. Pressure has value as a response biomarker, although measurement remains invasive or indirect and spatial heterogeneity complicates interpretation [66,68]. Approaches under study include stromal decompression, enzymatic reduction of hyaluronan or collagen, vasoactive or vascular normalizing regimens, ultrasound with microbubbles, mild hyperthermia, osmotic modulation, and carrier designs that are smaller, deformable, or transiently neutral to reduce pressure sensitivity [69,70,71]. Each option introduces trade-offs since matrix depletion can damage normal tissue, vascular normalization can narrow the permeability window, ultrasound produces uneven fields in fibrotic regions, and timing LNP dosing to short-lived pressure relief demands imaging guidance. Some cytotoxic or antiangiogenic therapies also remodel stroma in ways that increase stiffness and pressure, reinforcing barriers. Quantitative mapping of pressure and matrix architecture and integration with scheduling and carrier design are therefore central to achieving reliable intratumoral delivery of nucleic-acid therapeutics [59,66]. Beyond interstitial transport, another major hurdle lies inside the cell: most lipid nanoparticles fail to release their cargo once taken up. In fact, only a very small fraction—often cited as less than two percent—manages to escape the endosomal compartment and reach the cytoplasm where nucleic acids can act [72]. To overcome this inefficiency, several approaches have been pursued. Researchers have developed ionizable lipids that change charge in acidic environments and destabilize the endosomal membrane; others have tested fusion peptides inspired by viral proteins; and still others have explored photosensitizer-based systems that use light to trigger endosomal rupture [73,74]. These strategies are summarized in Table 5, which highlights their mechanisms, advantages, and major pitfalls—underscoring that each offers unique strengths but also carries significant limitations. None of these options is without trade-offs. Gains in delivery efficiency often come at the expense of safety, manufacturing robustness, or clinical practicality, which makes it unlikely that a single strategy will provide a universal solution [74].

### 4.3. Cellular Internalization Mechanisms

**Figure 6:**
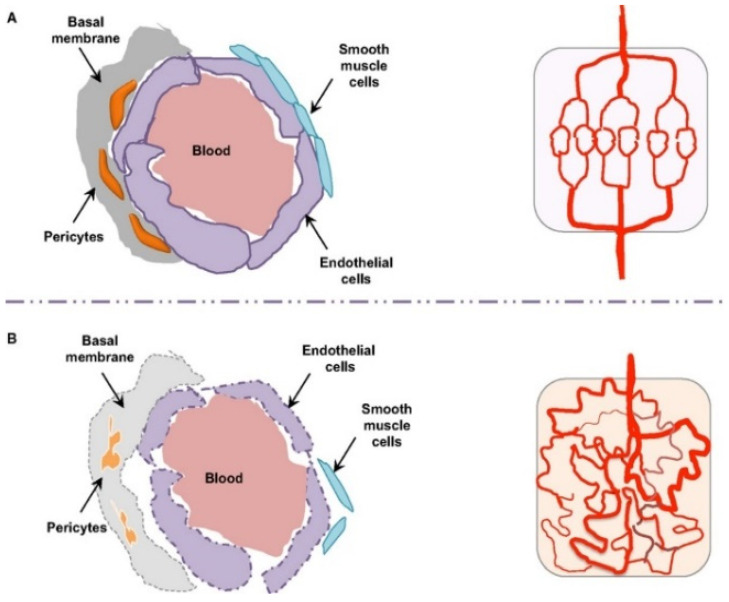
Endothelial barrier in normal and tumor vessels. The endothelial barrier structure differs in (**A**) normal and (**B**) tumor blood vessels. Compared with normal vessels, tumor vasculature is disorganized with loose endothelial junctions, aberrant pericytes, scarce smooth muscle, and altered basement membrane, features that influence nanoparticle extravasation and access to parenchymal cells.

Delivery of nucleic acids by lipid nanoparticles depends on how carriers enter cells and avoid lysosomal degradation, with the prevalence of specific routes shaped by cell type, ligand display, and the vascular context shown in Figure 6 [75]. Entry commonly proceeds through clathrin pathways, caveolae, macropinocytosis, or phagocytosis, and multiple routes can act in parallel within a single tissue [35]. Inferring mechanisms only from broad small-molecule inhibitors is unreliable because these agents perturb membranes and trafficking globally, so genetic perturbation and live imaging are favored to confirm design rules [75]. Carrier size, deformability, surface hydration, and charge bias receptor engagement and curvature sensing, and membrane-active or nonlamellar lipid phases can promote fusion-like entry that assists endosomal escape while risking colloidal instability and off-target interactions [35,75]. Targeting ligands enrich uptake but heterogeneous receptor expression and recycling can sequester particles at the periphery and divert cargo to degradation [76]. Stealth coatings extend circulation and reduce nonspecific binding while dampening contact-driven internalization, which motivates architectures that shed shields or reveal charge after extravasation in response to pH, redox, or protease cues [76,77]. Practical strategies pair ionizable lipids that activate within endosomes with reversible surface designs and dosing schedules aligned to tissue access, acknowledging that identical formulations may behave differently across tumor niches and normal organs and that mechanism mapping should accompany efficacy evaluation in relevant models [12].

## 5. Intracellular Delivery Determinants of Lipid Nanoparticles

### 5.1. Endocytic Pathways and Endosomal Sequestration

Lipid nanoparticles that deliver nucleic acids depend on cellular entry and timely cytosolic release, with internalization proceeding through clathrin-mediated, caveolae-mediated, and macropinocytic routes that can coexist within a single tissue [72,75]. Table 6 summarizes the principal features highlighted for mRNA-LNP uptake, noting clathrin-mediated uptake with about 100 nm clathrin-coated pits and receptor-specific rapid internalization, caveolae-mediated uptake through 50–80 nm cholesterol-rich flask-shaped invaginations often linked to cell-type selectivity, and macropinocytosis that enables nonselective bulk internalization from several hundred nanometers to micrometers [72]. After entry, most carriers remain confined to endosomes and are trafficked to lysosomes, where RNA cargo is degraded, and typical cytosolic delivery efficiencies remain below two percent [13]. Inefficiency is compounded by early dissociation of RNA from ionizable lipids inside endosomes, the fact that only a small subset of endosomes undergoes the membrane disruption marked by galectins, and rapid engagement of repair machinery such as ESCRT that reseals pores before escape [78]. Ionizable lipids acquire a positive charge in the acidic lumen and interact with anionic endosomal membranes to promote release, although the effective window is narrow and can be offset by cargo–lipid separation or unintended interactions that compromise colloidal stability [12]. These observations argue for designs that couple circulation stability with triggered endosomal transitions, including reversible surface shielding that exposes charge after extravasation, helper lipids that sustain fusion propensity without aggregation, and dosing schedules aligned with trafficking kinetics, alongside quantitative assays that localize where and when escape occurs rather than relying on broad inhibitor studies [79].

### 5.2. Endosomal Escape Mechanisms

**Figure 7:**
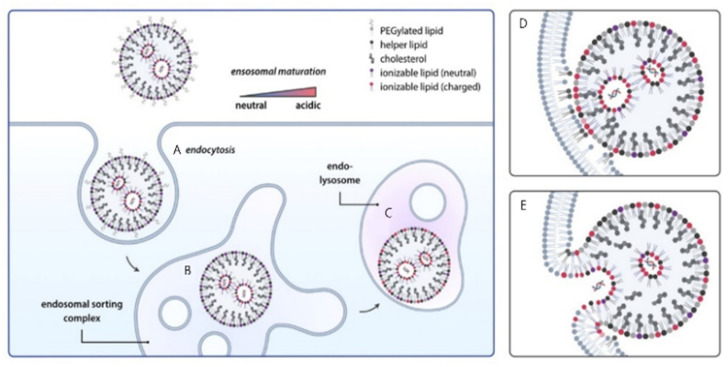
Schematic of endosomal escape by ionizable lipid nanoparticles. (**A**) endocytosis, (**B**) early endosome and sorting, (**C**) late endosome or endolysosome with acidification and protonation of ionizable lipids, (**D**) membrane engagement and fusion, (**E**) pore formation and cytosolic release. The diagram indicates PEGylated lipids, helper lipids, cholesterol, and ionizable lipids in neutral or charged states.

Cellular uptake of lipid nanoparticles is high while cytosolic delivery of nucleic acids typically remains below two percent because most carriers traffic from early to late endosomes and then to lysosomes where cargo is degraded [13]. Figure 7 illustrates the endocytic journey and progressive acidification that protonates ionizable lipids and promotes their engagement with anionic endosomal membranes, a sequence that can drive fusion and transient pore formation for release into the cytosol [80]. pH-responsive lipids with pKa values near 6 acquire positive charge in the endosome and, together with helper lipids and cholesterol, can induce nonlamellar transitions that lower the energetic barrier to fusion, although increased fusogenicity risks colloidal instability and membrane injury outside the target compartment [72,81]. Structural organization matters because inverted hexagonal and cubic tendencies favor escape compared with lamellar packing, but excessive disorder can impair circulation stability and trigger off-target interactions [82]. Only a small subset of endosomes appears permissive for release as indicated by galectin labeling, and rapid membrane repair through ESCRT can reseal pores before productive escape, while segregation of RNA from ionizable lipids inside maturing endosomes further lowers success [75,83]. Practical designs, therefore, aim to preserve cargo–lipid proximity during trafficking, program reversible PEG shielding and charge exposure after extravasation, and couple helper lipids or external stimuli such as light or heat in controlled regimens, acknowledging that indiscriminate perturbation can activate stress pathways and reduce viability. Figure 7 conveys these principles by depicting endocytosis, early endosome sorting, endolysosomal acidification, and lipid protonation that culminate in membrane fusion and nucleic acid release [81,84]. Our current picture of how lipid nanoparticles enter cells and escape from endosomes is built largely on results from static in vitro experiments [85]. While these systems are convenient, they represent only a narrow and simplified version of real biology. For example, a flat monolayer of cells cannot mimic blood circulation, the architecture of the extracellular matrix, or the complex crosstalk with stromal and immune cells [86]. They also miss the spatial heterogeneity that strongly shapes nanoparticle movement inside tissues [87]. Table 7 further illustrates these discrepancies, contrasting static in vitro conditions with the dynamic, heterogeneous features of in vivo environments that critically influence LNP performance. Because of this, measurements such as uptake rates or colocalization scores in cell culture often fail to match the pharmacokinetics, biodistribution, or therapeutic responses seen in living organisms. Bridging these gaps requires more than incremental improvement: it calls for critical reflection on how we interpret in vitro data and for systematic strategies that better align experimental models with in vivo performance [85,86].

### 5.3. Cytoplasmic Stability and Nucleic Acid Protection

**Figure 8:**
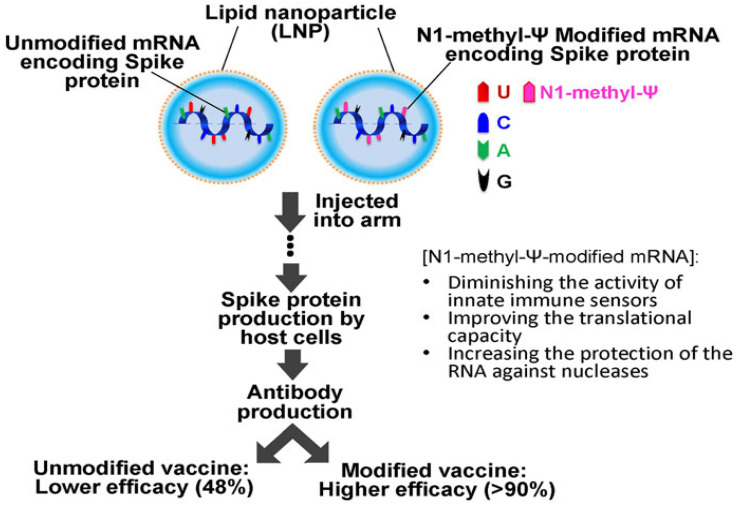
Schematic of LNP-mRNA vaccination. Modified nucleoside mRNA packaged in lipid nanoparticles shows reduced innate immune sensing, enhanced translation, and greater protection from nucleases, yielding higher efficacy than unmodified mRNA.

Following endosomal release, therapeutic mRNA encounters abundant cytosolic RNases and RNA quality control hubs, so the time window for productive translation is narrow and strongly influenced by RNA chemistry and lipid architecture [88]. Figure 8 illustrates that nucleoside modification with N1-methyl-pseudouridine within LNP-carried mRNA diminishes innate sensor engagement, improves translational capacity, and increases resistance to nucleases, which corresponds to higher vaccine efficacy compared with unmodified counterparts [89,90]. Stability is further tuned by 5′ cap optimization, polyadenylation length, and untranslated region engineering that together enhance ribosome loading while limiting innate activation [91]. LNP composition also governs fate after entry since ionizable lipids condense and shield RNA from RNases and cholesterol with helper phospholipids modulates core packing and membrane interactions, although excessive compaction can slow uncoating and reduce translation and highly cationic surfaces can trigger unwanted protein adsorption and inflammation [92]. An effective design maintains cargo proximity to ionizable lipids during trafficking, then enables timely decomplexation at ribosomes through pKa-matched ionizable lipids, degradable linkers, or responsive PEG shedding, acknowledging that over-suppressing innate sensing benefits gene replacement whereas vaccines may require some adjuvanticity [93]. Converging RNA chemistry with tunable LNP structure and manufacturing control provides a practical route to sustain cytoplasmic integrity and maximize protein expression without sacrificing safety [91,93].

### 5.4. Nuclear Transport Requirements for Plasmid DNA

Nuclear access remains the rate-limiting step for plasmid DNA delivered by nonviral carriers because the genome-sized cargo must traverse the cytoplasm, dock at the nuclear envelope, and cross a size-selective pore or exploit mitotic disassembly [94]. After cytosolic entry, plasmid complexes move along microtubules toward the perinuclear region through interactions between DNA-bound transcription factors and importin beta that recruit dynein and kinesin motors [95]. During mitosis, breakdown of the nuclear envelope permits chromosomal association and partitioning of DNA into daughter nuclei, which aligns with observations that reporter expression frequently appears after cell division and that incoming DNA is often encased by membrane fragments or tethered to chromosomes [94,96]. In nondividing cells, passage through nuclear pore complexes is uncommon and large complexes face stringent size and charge constraints [94]. Sequence-based tactics such as insertion of DNA nuclear targeting sequences have shown limited benefit in increasing nuclear copy numbers or transcription, indicating that nuclear delivery is governed by a broader set of variables [97]. Useful design levers include compacting plasmids to reduce hydrodynamic size while enabling timely unpacking, coupling cargos to karyophilic proteins or peptide nuclear localization signals, timing dosing to proliferative windows, and modulating microtubule engagement [98]. Each tactic involves trade-offs because stronger condensation can hinder transcriptional access, excessive NLS display can promote aggregation, and forced mitotic entry raises safety concerns, so success depends on integrating trafficking control with cell cycle considerations and intranuclear release [98].

## 6. Integrated Engineering and Translation of Lipid Nanoparticles for Nucleic Acid Delivery

### 6.1. Lipid Composition Engineering for LNP Delivery

#### 6.1.1. Ionizable Lipid Optimization

**Figure 9:**
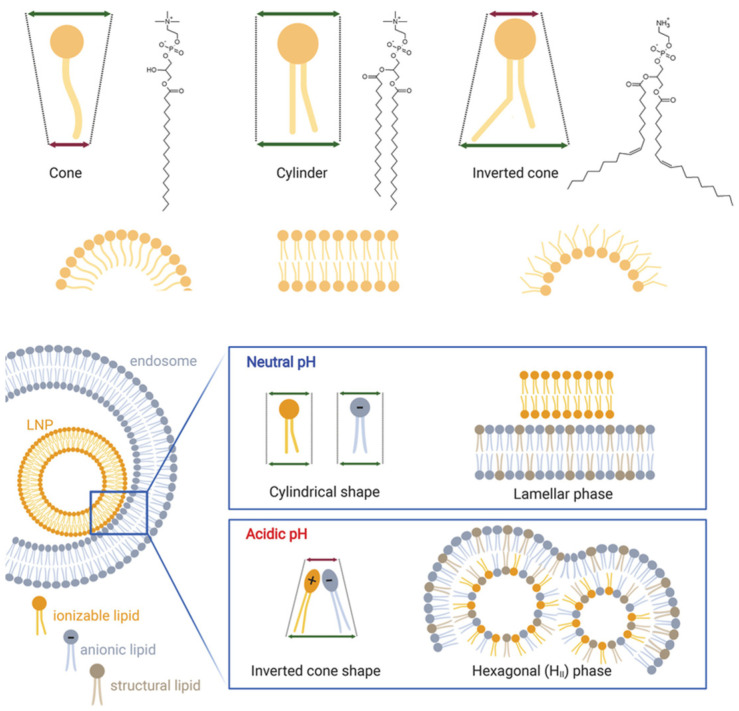
pH-responsive behavior of ionizable lipids and phase transitions that enable endosomal escape in lipid nanoparticles. At neutral pH, ionizable lipids are uncharged and favor lamellar organization within stable LNP cores. In the mildly acidic endosome, they become protonated, adopt inverted-cone geometry, and promote nonlamellar hexagonal HII phases that facilitate membrane fusion or pore formation and cytosolic release. The schematic indicates ionizable lipid, anionic lipid, and structural lipid components.

Ionizable lipids govern whether lipid nanoparticles achieve cytosolic delivery without provoking systemic toxicity by switching charge and packing as pH changes along the endocytic route [99]. At physiological pH, they remain largely neutral and adopt a cylindrical geometry that supports lamellar organization and circulation stability, whereas endosomal acidification increases protonation and drives an inverted cone geometry that favors nonlamellar transitions and membrane mixing as depicted in Figure 9 [100]. Performance depends not only on pKa near the mildly acidic range but also on headgroup sterics, linker chemistry, and hydrophobic tail architecture that together determine curvature stress, miscibility with helper lipids, and the balance between stability and fusogenicity [100]. Formulations that strongly promote hexagonal HII phases can improve escape while risking premature destabilization, protein adsorption, and hemolysis, and highly compacted cores shield RNA from nucleases while potentially delaying uncoating at ribosomes [101]. Designs that incorporate biodegradable ionizable lipids can reduce tissue retention, although metabolite profiles and durability of gene expression require careful evaluation [101]. Emerging architectures such as multication switching or double ionization motifs are promising for stronger endosomal engagement but may narrow the dosing window and raise safety concerns. Effective optimization, therefore, aligns pKa and molecular shape with trafficking kinetics, preserves cargo proximity to lipids during maturation, and enables timely decomplexation after release, accepting that gains in escape often trade off against colloidal stability and tolerability [102].

#### 6.1.2. Helper Lipid and Cholesterol Optimization

Helper phospholipids and cholesterol shape membrane packing, rigidity, and curvature in lipid nanoparticles and therefore influence circulation stability, protein corona formation, organ distribution, and endosomal release of nucleic acids [18]. Prior studies report that saturated phosphatidylcholines such as DSPC maintain particle morphology and limit premature leakage under physiological conditions, although the higher order they impose can suppress the negative curvature needed for membrane fusion during endosomal escape [17]. In contrast, helper lipids with unsaturation or phosphatidylethanolamine headgroups promote nonlamellar transitions that facilitate fusion and pore formation, while increasing the risk of aggregation and unintended membrane damage, which can lower tolerability [18]. Cholesterol fills packing defects and tunes membrane order and elasticity, but its fraction presents a narrow window because excessive amounts can dampen tumorgenicity and slow release whereas insufficient amounts compromise integrity and increase leakage [24]. Chemically modified cholesterol derivatives offer finer control of elasticity and phase propensity and can improve deformation during tissue passage, although shifts in pharmacokinetics and innate immune activation require monitoring [103]. An effective formulation co-optimizes helper lipid headgroups and acyl chains with cholesterol type and content to match trafficking and escape kinetics, accepting that improvements in endosomal release often trade off against colloidal stability and safety and therefore benefit from iterative testing in models that reflect the intended route and target tissue [2].

#### 6.1.3. PEG-Lipid Density and Shedding Control

PEG-lipids confer steric stabilization that limits opsonization and prolongs circulation, but their density, chain length, and anchor chemistry jointly determine how well lipid nanoparticles reach and engage target cells [18]. High surface coverage suppresses nonspecific protein binding and reticuloendothelial uptake while also creating a hydration barrier that reduces ligand access, membrane contact, and endosomal escape [23]. Low coverage improves interactions with cells but increases aggregation, complement activation, and rapid clearance [37]. The hydrophobic tail of the PEG-lipid governs desorption kinetics from the particle surface, where shorter or biodegradable anchors shed more readily after extravasation to expose charge or ligands, and longer anchors resist shedding and sustain stealth at the cost of weaker uptake [23]. Environmentally responsive designs that cleave PEG through hydrolysis, redox change, or mild acidity can reconcile these opposing needs when the trigger is synchronized with tissue access, although premature loss during circulation or incomplete removal in the target site undermines the benefit [37]. Repeated dosing introduces additional constraints because anti-PEG responses and accelerated clearance have been observed, which argues for careful control of PEG density and for evaluation of mixed stealth strategies such as combining PEG with zwitterionic or polyglycerol motifs [15]. Continued treatment not only speeds up clearance because of anti-PEG immunity, but it also makes long-term safety and effectiveness worse in chronic cases [26]. This event has caused many clinical projects to fail, which means that there should be different covert strategies [104]. Zwitterionic polymers, poly(2-oxazoline), polyglycerols, and degradable PEG analogs are some of the most recent attempts to create a stealth effect while making the immune system less likely to react [28]. Researchers are also looking into PEG designs that are temporary or can be cleaved, as well as mixed stealth coatings, to find a balance between stability in the first circulation and less ABC when redoing [105]. For LNP therapies to be effective in real-world applications, these strategies and vigilant monitoring of anti-PEG antibody titters are essential [106]. Effective optimization balances circulation stability with timely unmasking by tuning PEG length, surface fraction, and anchor composition to match trafficking and exposure windows while monitoring safety, immunogenicity, and reproducibility in models that reflect the intended route and organ target [37].

#### 6.1.4. Anti-PEG Immunity and Accelerated Blood Clearance

One of the biggest problems with moving PEGylated LNPs from the lab to the clinic is the development of anti-PEG immunity, which is shown by the accelerated blood clearance (ABC) phenomenon [26]. Many people already have anti-PEG antibodies, and taking more of them may make these immune reactions worse [27]. This poses a significant threat to clinical translation, particularly for chronic diseases requiring frequent or prolonged dosing, as subsequent doses of PEGylated nanoparticles often demonstrate modified biodistribution, reduced therapeutic efficacy, and diminished circulation half-lives [107]. This paradox highlights PEG’s dual function: it prolongs systemic circulation and provides steric stabilization, but it also induces immunogenicity that compromises sustained delivery [28,108]. The fact that ABC has already interrupted or ended a number of promising therapeutic programs, according to clinical observations, highlights how serious the problem is [109]. There have been several suggestions for how to make these problems less severe. Other stealth coatings that are less likely to cause an immune response, such as poly(2-oxazoline), polyglycerols, and zwitterionic polymers, have also been shown to extend circulation [110]. Also, degradable or cleavable PEG analogs that are shed after tissue extravasation can make it harder for antibodies to recognize the initial protection [109]. To find a balance between stable circulation and lower ABC levels after redosing, researchers are looking into mixed stealth coatings that use PEG and other polymers [111]. Additionally, optimized dosing regimens, decoy particles, and meticulous monitoring of anti-PEG antibody titters have been proposed as adjunctive strategies to mitigate the clinical effects of ABC. In general, PEGylation should be seen as both a way to make circulation more stable and a possible problem that limits its long-term use [110]. Future LNP development will benefit from systematic evaluation of anti-PEG immune responses, integration of alternative polymers, and adaptive dosing strategies to ensure sustainable clinical translation [112].

### 6.2. Tissue and Cell Targeting Strategies for Lipid Nanoparticles

#### 6.2.1. Hepatocyte-Directed Targeting

Hepatocyte-directed delivery builds on the liver’s propensity to clear nanoparticles while guiding carriers to productive uptake by parenchymal cells rather than sequestration by Kupffer cells or sinusoidal endothelium [48]. N-acetylgalactosamine ligands that engage the asialoglycoprotein receptor on hepatocytes enable efficient clathrin-mediated internalization and permit subcutaneous administration by maintaining targeting after systemic exposure, although performance depends strongly on ligand density, spatial orientation, and linker chemistry that governs receptor access and avidity [113]. Overcrowded presentation can create a steric canopy that limits membrane contact and slows endosomal escape, whereas sparse or weakly attached ligands are displaced by protein corona components and redirect particles to off-target sinks [113]. Endogenous pathways can be leveraged by tuning surface charge and lipid composition to promote apolipoprotein adsorption and low-density lipoprotein receptor engagement, which enhances hepatocyte uptake but increases sensitivity to biological variability in corona formation [114]. Design choices must balance affinity, pharmacokinetics, and intracellular routing because high-affinity binding can trap particles at the cell surface and favor recycling, and aggressive charge exposure improves uptake while elevating complement activation. Practical formulations, therefore, pair GalNAc or complementary motifs with controlled ligand spacing, cleavable linkers that unmask the surface after extravasation, and ionizable lipid cores that support endosomal escape, while accounting for disease-dependent changes in receptor expression and sinusoidal architecture that alter targeting efficiency across patient populations [113].

#### 6.2.2. Immune-Cell or Tumor-Specific Targeting

**Figure 10:**
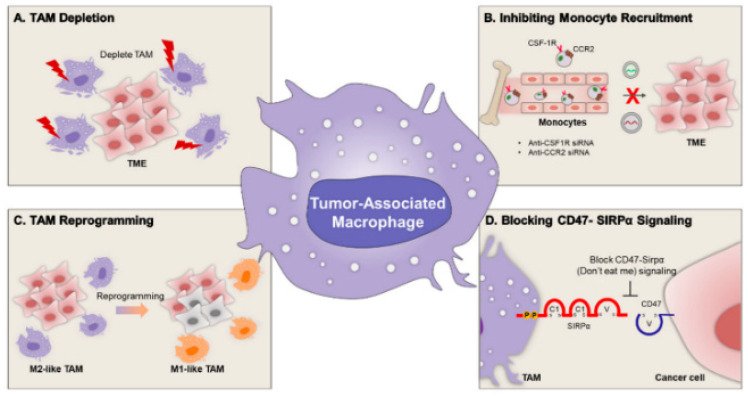
Ligand-directed lipid nanoparticle strategies for immune and tumor targeting. (**A**) Depletion of tumor-associated macrophages to reduce immunosuppression in the tumor microenvironment. (**B**) Inhibition of monocyte recruitment by blocking CSF-1R or CCR2 to limit macrophage replenishment. (**C**) Reprogramming of macrophages from M2-like to M1-like states to enhance antigen presentation and antitumor activity. (**D**) Blockade of CD47–SIRPα signaling to restore phagocytosis of cancer cells. Colors indicate different cell types and states: purple, tumor-associated macrophages (TAMs); orange, reprogrammed M1-like macrophages; red arrows, TAM depletion processes.

Active targeting of lipid nanoparticles to immune and tumor compartments seeks to convert heterogeneous accumulation into productive uptake by defined cell subsets while avoiding sequestration by the mononuclear phagocyte system [114]. Ligands such as antibodies, single-chain fragments, peptides, aptamers, or small molecules can be displayed to engage markers on dendritic cells, T cells, tumor-associated macrophages, or malignant cells, enabling receptor-mediated endocytosis and localized gene modulation as illustrated in Figure 10 [114]. Performance depends on how ligands are presented because density, spacing, and orientation determine receptor access and avidity, and excessive crowding or persistent PEG layers can hinder binding and dampen endosomal escape, whereas sparse coverage invites displacement by the protein corona and redirection to off-target sinks [18]. The tumor microenvironment introduces additional barriers including variable receptor expression, high interstitial pressure, and stromal shielding, which collectively lower the fraction of particles that reach intended cells and can bias trafficking toward lysosomal degradation [115]. Designs that use cleavable PEG, multivalent or bispecific constructs, pH or protease-responsive linkers, and locally administered or intratumoral dosing can improve engagement, although these same features may raise complement activation, accelerate clearance on repeat dosing, or increase immunogenicity if Fc domains or highly cationic surfaces are exposed [116]. Balancing affinity, pharmacokinetics, and intracellular routing while accounting for disease-dependent changes in myeloid composition and vascular integrity is therefore essential for reliable immune-cell or tumor-specific delivery by lipid nanoparticles [114].

#### 6.2.3. Organ-Selective Lipid Nanoparticles (SORT)

**Figure 11:**
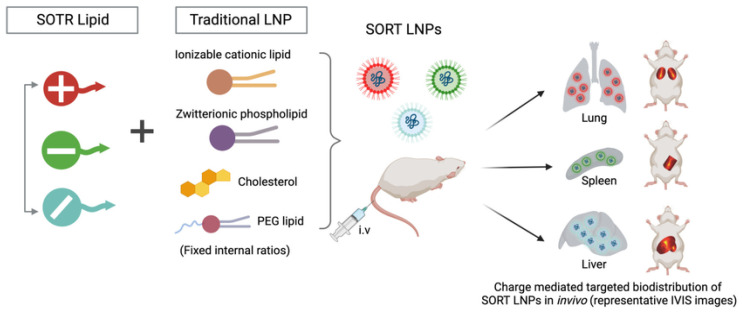
**Organ-selective delivery by selective organ targeting lipid nanoparticles (SORT).** Incorporation of specific SORT lipids into conventional LNPs alters surface properties and redirects tissue distribution. DOTAP promotes lung targeting, 18PA favors spleen accumulation, and DODAP retains liver delivery. Chemical structures of representative lipids used in the formulations are shown, including ionizable, helper, PEG, and SORT lipids.

Conventional lipid nanoparticles preferentially localize to the liver owing to sinusoidal fenestration, apolipoprotein adsorption, and efficient uptake by hepatocytes and resident phagocytes, so redirecting distribution requires control over surface chemistry without sacrificing stability [79]. Selective organ targeting modulates biodistribution by adding a small fraction of permanently charged or zwitterionic lipids during formulation, which shifts effective surface charge, alters protein corona composition, and biases receptor engagement, as summarized in Figure 11 [117]. Cationic additives can favor delivery to lungs or spleen, whereas specific anionic or zwitterionic components can increase access to lymphoid or marrow tissues, providing a modular route that does not depend on ligands [118,119]. This flexibility introduces trade-offs because small changes in identity or molar percent can move particles outside a narrow window for colloidal integrity, trigger complement activation, or reduce potency through excessive PEG shielding, and corona variability across sera can undermine predictability between models and patients [4]. Practical implementation, therefore, hinges on tight manufacturing control of additive ratios, orthogonal analytics that track corona profiles and charge in physiological media and pairing of SORT chemistry with endosomal escape capacity, so tissue retuning does not come at the cost of intracellular release [118]. Combining SORT with local dosing or conditional PEG shedding can further improve target engagement if toxicity, repeat-dose clearance, and disease-dependent vascular changes are evaluated in relevant models [98]. Although progress with SORT systems has been encouraging, their consistency across species and disease models is still uncertain [18]. Even subtle shifts in formulation, or changes in the protein corona that form in different biological fluids, can redirect organ tropism and make outcomes difficult to predict [120]. The addition of permanently charged lipids, while useful for returning biodistribution, also reduces the margin for colloidal stability and alters corona composition in ways that may affect uptake pathways and immune recognition [2]. To manage these risks, more careful analytical tools and testing in physiologically relevant settings are needed. For clinical translation, the most practical opportunities are likely in diseases where regional or catheter-based dosing can be applied, alongside SORT chemistries that integrate feasible endosomal-escape strategies. Reaching first-in-human studies will depend on strict control of formulation ratios and demonstration of reproducibility across multiple centers [18].

### 6.3. Endosomal Escape Engineering

#### 6.3.1. pH-Responsive Lipid Engineering

**Figure 12:**
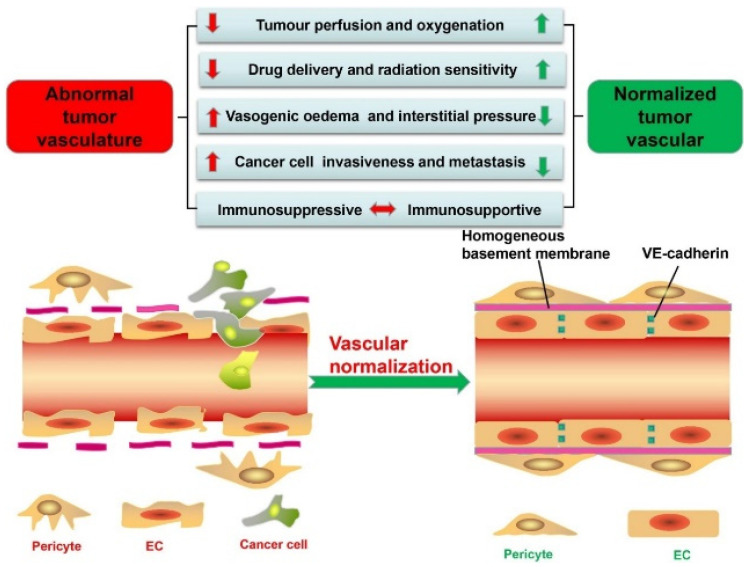
Abnormal tumor vasculature produces loose endothelial junctions, sparse pericyte coverage, and a discontinuous basement membrane that reduces perfusion, elevates interstitial pressure, and generates a hypoxic acidic microenvironment that suppresses therapy. Vascular normalization restores VE-cadherin adhesion, strengthens pericyte support, and rebuilds a homogeneous basement membrane, improving perfusion and oxygenation while lowering interstitial pressure and shifting the milieu toward immunosupportive conditions.

Ionizable lipids enable lipid nanoparticles to remain neutral at physiological pH for low systemic reactivity and to gain positive charge during endosomal acidification, where protonation strengthens interactions with anionic membranes and promotes fusion or transient pore formation that releases nucleic acids [121]. Delivery performance reflects not only pKa near the mildly acidic range of roughly 6 to 6.5 but also headgroup sterics, linker chemistry, and tail geometry that bias curvature and nonlamellar phase formation, with well-known lipids such as DLin-MC3-DMA illustrating this balance around pKa 6.4 [2]. These same features introduce trade-offs because increased fusogenicity can reduce colloidal stability, raise hemolysis risk, and trigger off-target membrane perturbation, while a narrow activation window and heterogeneous extracellular acidosis can lead to premature lipid protonation before cellular uptake [122]. The tumor milieu complicates matters further, as shown in Figure 12, where abnormal vasculature drives hypoxia and acidity that may favor activation but also limits perfusion, whereas vascular normalization improves oxygenation and raises pH, narrowing the window for pH-triggered release [123]. Effective designs, therefore, couple pKa-matched ionizable lipids with helper lipids that tune curvature without excessive leakage, preserve cargo proximity during trafficking, and allow timely decomplexation in the cytosol, with dosing schedules adapted to tissue physiology so gains in escape do not come at the expense of safety or exposure [2,122].

#### 6.3.2. Membrane-Fusion Peptides and Polymers

Cell-penetrating and fusogenic motifs are used to boost endosomal escape of lipid nanoparticle cargo by either promoting membrane merger or creating local osmotic and curvature stress that opens transient pores [124]. Representative peptides such as INF7, S19, and TAT fusions can undergo pH-triggered conformational changes in maturing endosomes that favor insertion and disruption, and cationic polymers including polyhistidine, protamine, and PAMAM enhance buffering and membrane destabilization while condensing nucleic acids [125,126]. These additions increase cytosolic delivery but introduce trade-offs that matter in practice because peptide density, orientation, and anchoring chemistry can impair colloidal stability, increase serum protein binding, and shift biodistribution, and strong membrane activity risks hemolysis, mitochondrial injury, and inflammation [126]. Quantitative essays that read out true cytosolic access such as SLEEQ report low escape fractions in common cell lines on the order of only a few percent, which argues for measured claims and for pairing these agents with ionizable lipid cores rather than relying on them as stand-alone solutions [127]. Strategies that mask charges with cleavable PEG, restrict activity to acidic or protease-rich niches, and use biodegradable polymer backbones can maintain circulation stability while enabling on-target activation, although incomplete triggering or premature unmasking reduces the benefit [125,128]. Effective designs therefore tune peptide or polymer loading and placement to complement, not overwhelm, the ionizable lipid mechanism, with escape gains weighed against safety, manufacturability, and reproducibility across relevant models [128].

### 6.4. Immunosuppression Strategies and Administration Pathways for Lipid Nanoparticles

#### 6.4.1. Incorporation of Immunosuppressive or Tolerogenic Agents

**Figure 13:**
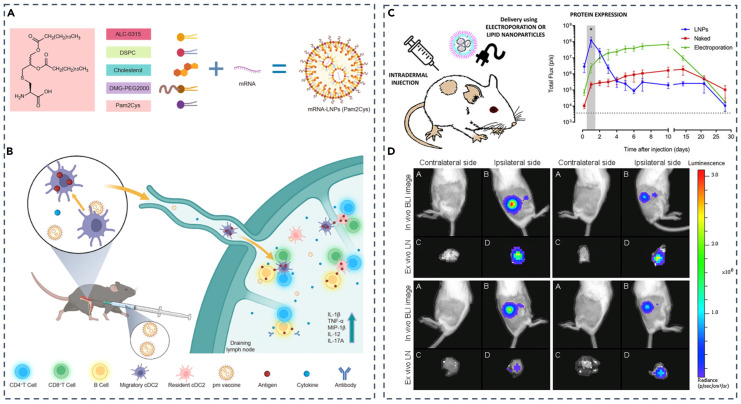
Lymph node targeting using lipid nanoparticles. (**A**) Formulation of Pam2Cys-incorporated mRNA-LNPs composed of ionizable lipid, DSPC, cholesterol, and PEG-lipid. (**B**) Concept of Pam2Cys-enhanced delivery to draining lymph nodes with engagement of resident and migratory dendritic cells and downstream adaptive responses. (**C**) Schematic of intradermal administration compared with electroporation and representative protein expression kinetics. (**D**) In vivo bioluminescence showing predominant expression in draining lymph nodes after intradermal saRNA-LNP injection. Taken from [114] with permission.

Lipid nanoparticles can be configured to dampen unwanted immunity or induce antigen-specific tolerance by co-delivering small molecules, protein or peptide antigens, and programmed mRNA while matching the formulation to the tissue reached and the cell types engaged [129]. Co-loading rapamycin with cognate antigen has been shown to bias dendritic cells toward regulatory programs and expand regulatory T cells, and nucleoside-modified mRNA reduces innate sensing to further curb reactogenicity [130]. Figure 13 illustrates a lymph node–addressable design in which Pam2Cys-incorporated mRNA-LNPs concentrate expression in draining nodes after intradermal dosing, supporting localized immune programming, while Table 8 summarizes how route selection shapes biodistribution and immune activation with intramuscular delivery driving systemic responses, intravenous delivery biasing hepatic expression with limited stimulation, and subcutaneous delivery focusing activity in draining lymph nodes [114]. These gains require balance because excessive immunosuppression risks infection, persistent PEG layers can hinder receptor access in nodes, and strongly membrane-active components that boost endosomal escape can reduce tolerability [131]. Practical regimens therefore tune cargo ratios, release kinetics, PEG shedding, and dosing route and verify antigen-specific T-cell and antibody outcomes rather than relying on surrogate readouts alone [114,130].

#### 6.4.2. Administration Routes and Dosing Strategies

Administration route and dose determine where lipid nanoparticles distribute, which antigen-presenting cells are engaged, and how strongly innate and adaptive programs are activated, making delivery planning part of the therapeutic mechanism rather than a neutral choice [132]. Intravenous delivery concentrates exposure in the liver through sinusoidal filtration and apolipoprotein interactions and suits hepatocyte targets while higher doses raise the risk of systemic reactogenicity [133]. Intramuscular injections produce strong local expressions with drainage to regional lymph nodes and measurable spillovers to liver and spleen, which supports broad immunity but increases off-target expression as dose escalates [134]. Subcutaneous or intradermal dosing focuses activity in draining lymph nodes and can be tuned toward tolerance with low repeated doses, although onset is slower and varies with injection depth and volume [134], whereas inhalation positions payloads in the airways where epithelial cells and phagocytes dominate uptake under constraints imposed by mucociliary clearance and irritancy [135]. Schedule further shapes outcomes because spaced low doses often improve T cell persistence and memory compared with a single large dose that heightens cytokines and complement and can saturate endosomal pathways, yielding diminishing returns [136]. Repeated administration introduces constraints including anti-PEG responses and accelerated clearance, which call for careful control of PEG density and shedding kinetics, interval selection, and premedication when appropriate [137]. Effective regimens align route, volume, and device with tissue access, pair dose with endosomal escape capacity to avoid trafficking bottlenecks and confirm antigen-specific function and safety in models that reflect disease-dependent physiology [132,134].

### 6.5. Manufacturing and Stability Engineering of Lipid Nanoparticles

#### 6.5.1. Microfluidic Mixing Parameters

Microfluidic synthesis allows precise control over the assembly of lipid nanoparticles for nucleic acid delivery, with performance tightly linked to how streams are combined and diluted [38]. The flow rate ratio, defined as the volumetric rate of the aqueous phase relative to the organic solvent, governs antisolvent dilution and nucleation; higher values usually drive rapid desolvation that produces smaller particles near the 30–40 nm range with low polydispersity, although excessive dilution can weaken core formation, lower encapsulation efficiency, and broaden size when neutralization is mistimed [138]. The total flow rate sets mixing time and Reynolds number; faster operation often narrows distributions but raises shear and pressure, increases heat generation, and can aggravate channel fouling or stress-sensitive RNA [139]. Intensified micromixer geometries such as staggered herringbone and vortex-inducing designs improve reproducibility and process stability while introducing device variability and cleaning validation burdens during scale-up [138,140]. Composition and environment intersect with hydrodynamics because of lipid concentration, ethanol fraction, buffer pH and ionic strength, and temperature shift assembly pathways and endow different packing states; cooler or low-solvent conditions can yield larger particles through slower lipid rearrangement, whereas moderate warmth increases membrane mobility at the risk of cargo degradation [141]. Studies that map delivery against flow conditions show that an FRR–TFR pair that maximizes in vivo potency does not always coincide with the smallest size or highest encapsulation, underscoring that mixing must be tuned with the intended route, endosomal escape capacity, and stability constraints [142]. Practical implementation, therefore, combines tight control of FRR and TFR with at-line analytics, robust device qualification, and numbering-up strategies that preserve residence time distributions, accepting trade-offs between colloidal stability, encapsulation yield, and manufacturability under GMP [38,139].

#### 6.5.2. Lyophilization, Storage, and Long-Term Stability

Long-term stabilization of lipid nanoparticles for nucleic acid delivery depends on controlling chemical degradation of lipids and RNA and preventing physical instabilities that appear as growth in hydrodynamic size, loss of encapsulation, or altered surface charge during storage [143]. Lyophilization with disaccharide protectants such as sucrose or trehalose preserves structure by vitrifying the matrix and replacing water at the membrane interface, which maintains particle integrity through freezing and drying and allows reconstitution with minimal change in size distribution when excipient to lipid ratios and residual moisture are optimized [144]. Benefits are not automatic because buffer species can crystallize during freezing and drive pH excursions that accelerate RNA hydrolysis and ionizable lipid breakdown, and rapid primary drying or overly warm secondary drying can collapse the cake and impair redispersion [145]. Storage at refrigerated temperature after successful freeze-drying supports retention of mRNA translation capacity and colloidal properties, although oxygen ingress and trace metals promote oxidation, which argues for oxygen-barrier packaging, judicious emissions, and avoidance of reactive headspace [146]. Formulation variables intersect with process settings since ethanol carryover, lipid composition, and nucleic acid length influence glass transition and protectant needs, and reconstitution medium, ionic strength, and temperature determine whether aggregates form on thawing [147]. Practical development, therefore, selects buffers with stable freezing behavior, sets conservative primary and secondary drying endpoints based on product temperature and glass transition, targets low residual moisture that still permits rapid redispersion, and confirms stability under accelerated and in-use conditions including repeated needle aspiration and simulated transport [144,147]. These controls expand distribution beyond ultracold chains and reduce costs while acknowledging trade-offs because higher sugar loads increase tonicity and prolonged drying can damage cargo, so process parameters must be tuned alongside chemistry to deliver durable and readily reconstituted lipid nanoparticle therapeutics [144,148]. Turning a laboratory concept into a product that can be manufactured under GMP conditions is never simple. Once production is scaled up, critical quality attributes (CQAs) such as particle size, RNA integrity, encapsulation efficiency, and surface charge can drift, sometimes enough to affect safety or performance [149]. Keeping these factors under control requires more than optimized mixing or improved stabilization. It also depends on real-time analytics, clearly defined release standards, and early integration with regulatory frameworks [150]. Without these safeguards, even the most promising designs may remain impressive in the lab but fail to advance toward clinical or commercial use [149,150].

## 7. Next-Generation LNP Systems and Precision Therapeutic Design

### 7.1. Hybrid and Multifunctional Nanocarriers Integrating Lipid Nanoparticles

**Figure 14:**
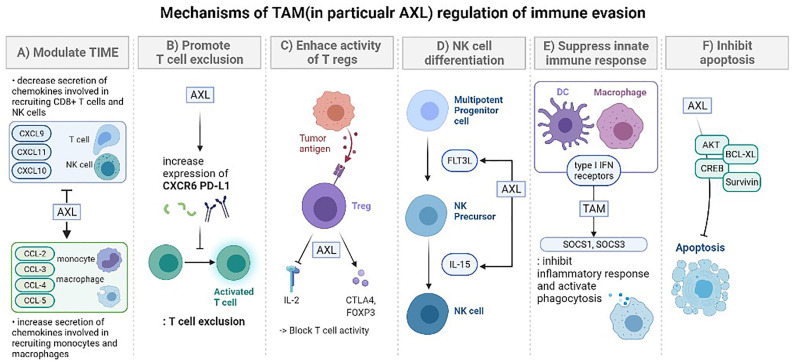
AXL signaling and immune evasion mediated by tumor-associated macrophages. (**A**) Modulation of the tumor immune microenvironment through chemokine shifts that reduce recruitment of CD8+ T cells and NK cells. **(B**) Promotion of T-cell exclusion with increased CXCR6 and PD-L1 expression. (**C)** Enhancement of regulatory T-cell activity that suppresses effector function. **(D**) Impairment of NK-cell differentiation via altered FLT3L and IL-15 pathways. (**E**) Suppression of innate immune responses through SOCS proteins affecting dendritic cells and macrophages. (**F**) Inhibition of apoptosis via AKT, CREB, BCL-XL, and survivin. These pathways underscore delivery targets for hybrid LNP systems aimed at macrophage depletion or reprogramming and restoration of antitumor immunity.

Hybrid carriers that combine polymer and lipid domains seek to extend the capabilities of single-component nanoparticles for nucleic acid delivery by uniting polymeric condensation and mechanical robustness with lipid-enabled biocompatibility, shielding, and targeting [151,152]. Core–shell architectures such as lipid–polymer hybrids and solvent-free lipid nanocapsules can co-load mRNA, siRNA, or plasmid DNA with adjuvant small molecules, present ligands for cell-type selectivity, and incorporate stimuli-responsive elements that time uncoating and release [153,154,155]. Polymer cores improve packing and protect long nucleic acids, whereas lipid shells reduce opsonization and enable receptor engagement, and charge-tunable designs aid endosomal interaction [151,152,156]. These advantages come with practical trade-offs that matter in translation because added interfaces complicate reproducible manufacturing, component incompatibilities can destabilize colloids during storage, and strong cationic elements raise complement activation and off-target uptake [15,151]. Mechanistic readouts of true cytosolic access remain scarce, so claims of improved escape require direct quantification rather than reliance on inhibitor studies [69]. Figure 14 highlights how tumor-associated macrophages leverage AXL signaling to suppress cytotoxic lymphocytes, expand regulatory T cells, and blunt innate responses, which motivates hybrid systems that deliver siRNA, CRISPR cargo, or kinase inhibitors to deplete or reprogram these cells while preserving delivery to the tumor or stimulating targets [157,158,159]. Viewed together, multifunctional hybrids offer a route to higher payload capacity and programmable biology provided that design choices balance potency with manufacturability, long-term stability, and safety across disease-relevant models [15,150,151].

### 7.2. Personalized and Precision Nanomedicine Approaches

Personalized use of lipid nanoparticles aims to match carrier design to patient-specific tumor microenvironments where extracellular matrix density, pH, protease activity, interstitial pressure, and receptor display vary widely and change over time [115]. Modular surfaces that present ligands against integrins or folate receptors can increase binding and uptake while multi-stage constructs that shrink in size, expose charge, or reveal ligands in response to acidity, redox cues, or proteases improve penetration and intracellular access [160]. These ideas promise higher precision but introduce trade-offs because dense ligand coating creates steric barriers, stable PEG layers can mask targeting motifs, protein corona remodeling can redirect tropism, and multiple triggers complicate manufacturing and quality control [161,162]. A practical pathway is to define a small set of microenvironment phenotypes and pair each with streamlined LNP variants that balance affinity with stealth, employ reversible PEG architectures, and use ionizable lipids tuned for endosomal escape under the expected pH range [115,160]. Selection and dosing can be guided by companion biomarkers from imaging, liquid biopsy, or ex vivo patient-derived models that report matrix stiffness, enzyme signatures, and receptor abundance, with schedules adjusted to windows of lowered interstitial pressure or vascular normalization. Such patient-tailored strategies treat LNPs not only as carriers but as adaptable platforms whose composition, targeting, and regimen are selected to the biological context while maintaining manufacturability and safety [115,163].

### 7.3. Marketed Lipid Nanocarrier-Based Delivery Platforms

The fact that lipid nanocarrier technologies have been successfully turned into treatments that are available for sale shows that they work in the clinic and have sped up the creation of nucleic acid-based drugs [164]. In the past ten years, regulators have approved several formulations, setting standards for safety, effectiveness, and the ability to be made [164]. Table 9 gives a brief overview of some examples.

The first siRNA treatment authorized by the FDA to be administered via LNPs was Onpattro^®^ [165]. For gene silencing technologies, this was a significant advance. As demonstrated by the rapid development and widespread use of mRNA vaccines such as Spikevax^®^ and Comirnaty^®^, LNP systems can be adapted to meet pressing public health needs [166]. LNP-based platforms are still being used to treat rare and long-term disorders, as shown by Irdelisiran and other late-stage clinical candidates [91]. These products you can buy are examples of the design ideas discussed in this review and show how flexible lipid nanocarrier engineering can be [167]. Their successes show that we need to make PEG-lipid dynamics, ionizable lipid chemistry, and manufacturing processes better in order to create drugs that are safe and effective for a large number of people [166].

## 8. Conclusions

Lipid nanoparticles have significantly improved the delivery of nucleic acids by integrating scalable formulation with adjustable interactions throughout the bloodstream, endothelium, extracellular matrix, and intracellular compartments; however, their effectiveness is still limited by associated barriers. Successful designs harmonize ionizable lipid pKa and curvature with endosomal pH, balance helper lipids and cholesterol to regulate membrane order and fusion potential, and adjust PEG density and shedding to address the balance between stealth and engagement. The use of targeting tactics such as hepatocyte ligands, immune or tumor selectivity, and compositional adjustments for organ tropism expands the focus beyond the liver. Additionally, escape enhancers and nucleic acid chemistries enhance cytosolic access and translation. A small fraction of endosomes facilitates effective release, the makeup of the protein corona differs depending on the tissue and disease, and repeated dosing may hasten clearance. Therefore, improvements in potency need to be balanced with considerations of tolerability and longevity.

Advancement relies on connecting formulation decisions to quantifiable biological results in scenarios that mirror clinical application. The focus is on standardized assays that accurately measure cytosolic delivery and, when applicable, nuclear entry. Additionally, it highlights the importance of route and schedule designs that align with vascular and interstitial limitations, along with production controls that link microfluidic mixing parameters to in-process analytics and stable formulations achieved through lyophilization. Identifying microenvironmental traits for patient categorization and linking them to the most effective LNP variants can enhance precision, as long as long-term safety, immunogenicity, and consistency across various species and disease conditions are confirmed. The integration of rational chemistry, quantitative biology, immunological modulation, and GMP-compliant methods enables the expansion of LNP treatments beyond liver targets, ensuring consistent benefits across various tissues and patient populations. The lipid-based nanoplatform needs to be reborn as a module system capable of integrating composition, structure, and function in order to obtain customized treatment results, not just as an inert carrier. siRNA therapy and mRNA vaccines have been proven through their impact on modern medicine and clinical validation. Advances such as long-term selective lipid formulation, stimulation-responsive design, PEG replacement, and AI-assisted design are expected to solve persistent intracellular and extracellular problems. These developments aim at accurate and consistent delivery, patient-centered treatment, and improved and strict quality assurance. By combining translation methods and mechanistic understanding, we can expect the advancement of precision nucleic acid nanomedicine in LNPs.

## 9. Patents

Several recent patents have had an impact on the creation of lipid-based colloidal carriers for delivering nucleic acids. This shows that LNP technology is moving from the lab to the clinic. Table 10 gives an overview of some examples.

These patents demonstrate the increasing complexity of lipid nanoparticle technologies. They discuss how developing new ionizable lipids, refining formulations, and delivering multifunctional payloads can enhance manufacturing. Their inclusion demonstrates the critical role that intellectual property plays in the development of nucleic acid nanomedicine.

## Figures and Tables

**Table 1:** Barrier-by-Barrier Engineering Framework for LNP Design.

Barrier	Key Challenge	Representative Strategies	Advantages	Limitations/Trade-offs
Circulation	Rapid clearance, opsonization	PEGylation, zwitterionic coatings	Prolonged circulation, stealth effect	Anti-PEG immunity, reduced uptake efficiency
Protein corona	Ligand masking, altered biodistribution	Pre-coating, corona engineering	Potential for controlled tropism	Variability across species/patient
Vasclar/ECM penetration	High interstitial pressure, dense matrix	Enzyme co-delivery, deformable/neutral particles	Improved tissue penetration	Risk of off-target toxicity, tissue remodeling
Cellular uptake	Heterogeneous endocytosis routes	Ligand conjugation, receptor targeting	Higher uptake in specific cells	Corona masking, receptor downregulation
Endosomal escape	<2% efficiency into cytoplasm	Ionizable lipids, fusion peptides, photosensitizers	Enhanced cytosolic release	Safety issues, poor scalability, limited reproducibility
Cytoplasmic stability	Nuclease degradation, innate immunity	Nucleoside modification, shielding polymers	Extended RNA stability	May alter translation efficiency
Nuclear access (DNA)	Nuclear envelope barrier	Nuclear localization signals, mitosis coupling	Enables plasmid/CRISPR delivery	Low efficiency, cell cycle dependence

**Table 2:** Core lipid components of lipid-based nanoparticles and their roles, mechanistic contributions, and common limitations in nucleic acid delivery.

Component	Function in LNP	Structural Role	Notes and Limitations
**Ionizable lipids**	Encapsulation and endosomal escape	Neutral at physiological pH, protonates in endosomes to disrupt membranes	Apparent pKa, tail architecture, and linker design require tuning for efficacy and safety
**Helper** **phospholipids**	Bilayer stability and fusion support	Maintain lamellar structure and reduce leakage	Highly ordered high-Tm species can impede fusion and slow release
**Cholesterol**	Membrane packing and fluidity modulation	Adjusts rigidity and can promote nonlamellar phases	Excess may stiffen membranes and alter organ tropism
**PEG-lipids**	Stealth and prolonged circulation	Steric shielding that reduces opsonization	High surface density or slow deshielding can hinder uptake and endosomal escape

**Table 3:** Physiological barriers affecting lipid-based nanoparticle delivery of nucleic acids with key challenges, major influencing factors, and representative engineering strategies.

Barrier	Key Challenges	Major Influencing Factors	Representative Strategies
**Hemodynamic shear and** **dilution**	Shear-induced instability, dilution upon injection, reduced vascular residence	Particle size, surface charge, PEG-lipid density	Optimize PEG density, use near-neutral surface charge, size tuning
**Protein corona formation and opsonization**	Non-specific protein binding, rapid MPS clearance, loss of targeting ability	Surface chemistry, PEGylation, lipid composition	PEGylation, zwitterionic/bioinspired coatings, controlled corona engineering
**RES (MPS) clearance**	Phagocytic uptake by liver/spleen macrophages, reduced delivery to target tissues	Size > 150 nm, cationic charge, protein corona	Size optimization (<150 nm), PEG stealth layer, decoy particles, immune-inert coatings
**Vascular** **endothelial barriers**	Limited extravasation into target tissues, especially in non- fenestrated organs	Tissue-specific endothelium, particle deformability, ligand use	EPR effect exploitation (in tumors), receptor-mediated transcytosis, active targeting ligands

(MPS, mononuclear phagocyte system; RES, reticuloendothelial system; EPR, enhanced permeability and retention.).

**Table 4:** Vascular endothelial phenotypes across tissues and qualitative extravasation potential of lipid-based nanoparticles with key delivery remarks.

Tissue Type	Endothelial Type	Fenestration/ Permeability	LNP Extravasation Potential	Remarks
**Liver** **(sinusoid)**	Fenestrated, discontinuous	High	High	Rapid uptake by Kupffer cells
**Spleen**	Fenestrated, slit-like pores	Moderate	Moderate	Filtration-based selectivity
**Tumor**	Leaky, disorganized vasculature	Very high (EPR effect)	High (but heterogeneous)	Interstitial pressure hinders deep delivery
**Normal** **tissue**	Continuous, tight junction	Low	Low	Minimal passive permeability

**Table 5:** Comparative evaluation of major strategies for enhancing endosomal escape.

Strategy	Mechanism of Action	Advantages
Novel ionizable lipids	Protonation at acidic pH → membrane destabilization	Tunable chemistry, high efficiency
Fusion peptides	Viral-mimetic membrane fusion and pore formation	Potent endosomal release, biomimetic mechanism
Photosensitization	Light-activated ROSs disrupt endosomal membranes	Spatiotemporal control, efficient release

**Table 6:** Major endocytosis pathways relevant to lipid nanoparticle uptake with size and structural features and characteristic behaviors of mRNA-LNP internalization.

Endocytosis Pathway	Size & Structural Features	Characteristics of mRNA-LNP Internalization
**Clathrin-mediated** **(Receptor-mediated)**	~100 nm, clathrin-coated pits	Receptor-specific uptake; rapid and efficient internalization
**Caveolae-mediated**	50–80 nm, caveolin-enriched flask-shaped invaginations	Cholesterol-rich; involved in certain cell-specific uptake
**Macropinocytosis**	Several hundred nm to micrometers	Non-selective bulk uptake; important in large-volume internalization

**Table 7:** Key Differences Between In Vitro Static Cell Culture Models and In Vivo Environments Relevant to LNP Performance.

Aspect	In Vitro (Static Cell Culture)	In Vivo Environment
Fluid dynamics	Static media, no flow	Shear stress, blood/lymph flow, dynamic perfusion
Extracellular matrix	Minimal or absent	Dense, heterogeneous ECM impeding penetration
Cellular composition	Single or limited cell types	Multiple cell types incl. stromal, endothelial, immune
Protein corona	Often absent or serum- supplement artifact	Dynamic, biofluid-specific coronas altering uptake
Readouts	Uptake %, colocalization, reporter expression	Biodistribution, pharmacokinetics, immune clearance

**Table 8:** Tissue Distribution and Immune Response Patterns of Lipid Nanoparticles (LNPs) According to Administration Routes.

Administration Route	Primary Tissue Distribution	mRNA Expression & Immune Activation Pattern
**IM (Intramuscular)**	Muscle, lymph nodes, liver, spleen	Induces systemic immune responses; strong CD8^+^ T-cell activation
**IV (Intravenous)**	Primarily liver, spleen (RES)	Dominant hepatic uptake; limited immune stimulation
**SC (Subcutaneous)**	Draining lymph nodes	Specialized for local lymph node immune activation

**Table 9:** Representative marketed lipid nanocarrier-based nucleic acid delivery platforms.

Product Name	Company/Developer	Nucleic Acid Cargo	Indication	Approval Year
Onpattro® (patisiran)	Alnylam Pharmaceuticals	siRNA	Hereditary transthyretin-mediated amyloidosis (hATTR)	2018
Comirnaty® (BNT162b2)	Pfizer/BioNTech	mRNA	COVID-19 vaccine	2020
Spikevax® (mRNA-1273)	Moderna Therapeutics	mRNA	COVID-19 vaccine	2020
Irdelisiran (ALN-TTRsc02, in late-stage trials)	Alnylam Pharmaceuticals	siRNA	Transthyretin amyloidosis	Ongoing (Phase III)

**Table 10:** Representative patents on lipid-based colloidal carriers for nucleic acid delivery.

Patent Number	Jurisdiction	Year	Key Focus	REF
US 11951177 B2	USA	2024	High-sterol LNP formulations for nucleic acid delivery	[168]
US 20230097090 A1	USA	2023	Multipurpose LNPs for diverse nucleic acids, including CRISPR cargo	[169]

## Data Availability

Not applicable.

## Abbreviations

The following abbreviations are used in this manuscript:

EPR	Enhanced Permeability and Retention
FRR	Flow Rate Ratio
IM	Intramuscular
IV	Intravenous
LNP	Lipid Nanoparticle
MPS	Mononuclear Phagocyte System
NLS	Nuclear Localization Signal
PEG	Polyethylene Glycol
RES	Reticuloendothelial System
SC	Subcutaneous
SORT	Selective Organ Targeting
TAM	Tumor-Associated Macrophage
TFR	Total Flow Rate
TME	Tumor Microenvironment
